# Identification of Hepatic Niche Harboring Human Acute Lymphoblastic Leukemic Cells via the SDF-1/CXCR4 Axis

**DOI:** 10.1371/journal.pone.0027042

**Published:** 2011-11-01

**Authors:** Itaru Kato, Akira Niwa, Toshio Heike, Hisanori Fujino, Megumu K. Saito, Katsutsugu Umeda, Hidefumi Hiramatsu, Mamoru Ito, Makiko Morita, Yoko Nishinaka, Souichi Adachi, Fumihiko Ishikawa, Tatsutoshi Nakahata

**Affiliations:** 1 Department of Clinical Application, Center for iPS Cell Research and Application, Kyoto University, Kyoto, Japan; 2 Department of Pediatrics, Graduate School of Medicine, Kyoto University, Kyoto, Japan; 3 Centre for Stem Cell Research, Brown Foundation Institute of Molecular Medicine for the Prevention of Human Diseases, The University of Texas Health Science Center at Houston, Houston, Texas, United States of America; 4 Central Institute for Experimental Animals, Kanagawa, Japan; 5 Human Health Sciences, Graduate School of Medicine, Kyoto University, Kyoto, Japan; 6 Research Unit for Human Disease Models, RIKEN Research Center for Allergy and Immunology, Kanagawa, Japan; Clinica Universidad de Navarra, Spain

## Abstract

In acute lymphoblastic leukemia (ALL) patients, the bone marrow niche is widely known to be an important element of treatment response and relapse. Furthermore, a characteristic liver pathology observed in ALL patients implies that the hepatic microenvironment provides an extramedullary niche for leukemic cells. However, it remains unclear whether the liver actually provides a specific niche. The mechanism underlying this pathology is also poorly understood. Here, to answer these questions, we reconstituted the histopathology of leukemic liver by using patients-derived primary ALL cells into NOD/SCID/Yc ^null^ mice. The liver pathology in this model was similar to that observed in the patients. By using this model, we clearly demonstrated that bile duct epithelial cells form a hepatic niche that supports infiltration and proliferation of ALL cells in the liver. Furthermore, we showed that functions of the niche are maintained by the SDF-1/CXCR4 axis, proposing a novel therapeutic approach targeting the extramedullary niche by inhibition of the SDF-1/CXCR4 axis. In conclusion, we demonstrated that the liver dissemination of leukemia is not due to nonselective infiltration, but rather systematic invasion and proliferation of leukemic cells in hepatic niche. Although the contribution of SDF-1/CXCR4 axis is reported in some cancer cells or leukemic niches such as bone marrow, we demonstrated that this axis works even in the extramedullary niche of leukemic cells. Our findings form the basis for therapeutic approaches that target the extramedullary niche by inhibiting the SDF-1/CXCR4 axis.

## Introduction

Acute lymphoblastic leukemia (ALL) is the most common malignant disease in children[1]. Although accumulated improvements in treatment regimens have raised the 5-year survival rate to as high as 80% in pediatric patients[2], a poor prognosis is still expected for a minority of patients with various risk factors and those with ALL relapses. In particular, relapsed ALL has an overall survival rate of only 30%[3].

Recent studies about leukemic cells and niche correlation highlight the importance of therapeutically targeting the bone marrow (BM) microenvironment [4], [5]. The BM niche provides survival and growth factors for leukemic cells, modulates their responses to chemotherapies and may even contribute to the relapse of the disease. But little is known about the extramedullary niche of leukemia.

Organ involvement varies with the type of neoplastic cell[6]. Such cells find their own appropriate microenviromental conditions in particular tissues for survival and proliferation[6]. The widespread involvement of extramedullary organs is characteristic of leukemia. Although leukemic cells can easily disseminate to all organs by traveling in the peripheral blood, the most striking changes are restricted in organs such as the liver and spleen. Even after leukemic cells seem to disappear after treatment, residual leukemic cells are thought to be released from BM and extramedullary niche, eventually causing recurrence of the disease [7]. Consequently, investigation of the interactions between leukemic cells and the niche at extramedullary sites is a crucial component in the management and overcome of leukemia; however, little is known about the role of extramedullary niche in harboring leukemic cells. Specifically, whether extramedullary sites actually provide a specific niche, and the factors responsible for harboring leukemic cells in extramedullary niche remain unclear.

We previously developed a novel immunodeficient NOD/SCID/Yc ^null^ (NOG) mouse that provides significantly better human hematopoietic cell engraftment in the BM and extramedullary organs than other immunodeficient mice[8], [9], [10], [11], and is capable of supporting the growth of human neoplastic cells[12]. In the present study, we introduced a human leukemic mouse xenograft model using the NOG mice attended by extramedullary involvement without pre-conditioning (hereby referred to as the h-leukemic NOG model). Previous animal xeno-transplantation models for recapitulating human leukemia required preconditioning to avoid graft rejection [13], [14]. However, these models are not appropriate for detailed pathological assessment of extramedullary microenvironments because preconditioning causes modification of the microenvironment such as upregulation of SDF-1 [15]. As the h-leukemic NOG model can reproduce leukemic extramedullary involvement without preconditioning, our approach provides a more sophisticated and physiological model suitable to study the interactions between leukemic cells and the host niche.

Leukemic cells preferentially infiltrate the liver, and the hepatomegaly is detected in as high as 30–50% of acute lymphoblastic leukemia (ALL) patients at diagnosis[16]. As hepatopathology of ALL is well characterized[17], but little is known about the molecular mechanisms that contribute to this pathology, we have focused on the liver for studying the role of extramedullary niche in sustaining leukemia.

In this paper, we have demonstrated that hepatomegaly and pathology in ALL patients are not only due to random infiltration but rather the result of SDF-1/CXCR4 axis-dependent migration and expansion of leukemic cells in the hepatic niche. Moreover, we have succeeded in suppressing post-chemotherapeutic leukemic regrowth in the hepatic niche by inhibiting the SDF-1/CXCR4 axis, thus resulting in an improvement in the overall survival. Although the contribution of SDF-1/CXCR4 axis is reported in some cancer cells [18], [19] or leukemic niches such as bone marrow [20], we demonstrated that this axis works even in the extramedullary niche of leukemic cells. These results provide a better understanding of the mechanisms of the extramedullary dissemination and aid in the development of ALL therapies that target the extramedullary niche.

## Results

### Human leukemic cells can be serially reconstituted in NOG mice without pre-conditioning

**Figure 1:**
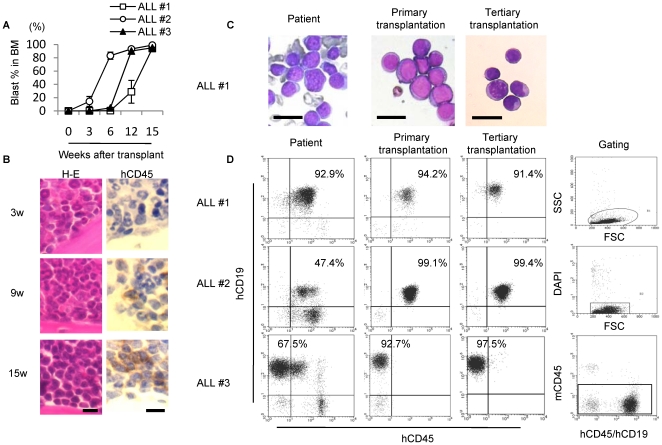
Leukemic cells can be engrafted to NOG mice without pre-conditioning. Morphology ( May-Grunwald Giemsa staining) and immunophenotype of leukemic blasts remain stable during serial transplantation in NOG mice. (A) Sequential FACS analyses showing bone marrow engraftment after transplantation. Graphs show percentage of blast cells (hCD45 or hCD19 positive cells) in recipient BM (*n* = 3−6 mice per case).Data are shown as means±S.D. (B) H-E staining and anti-hCD45 immunostaining showing increasing number of leukemic cells over time in the humerus of ALL#1 leukemic cell-recipient NOG mice. Scale bar, 10 µm. (C) Morphology and (D) immunophenotype of original patient blast cells (left row) and BM samples derived from murine primary and tertiary transplants of leukemic cells (middle and right rows). Debris (low forward scatter), dead cells (DAPI-positive), and mouse CD45 positive cells were excluded from analysis. Scale bar, 20 µm.

To establish a xenograft murine model of human ALL without damage to the recipient niche (h-leukemic NOG model), we first injected 1×10^6^ leukemic cells derived from primary BM of nine ALL children into the tail veins of untreated NOG mice (Table 1). Flow cytometric analysis of the BM revealed that eight of the nine leukemias engrafted in transplanted mice were detectable three weeks post-transplantation, and that BM chimerisms reached more than 40% of leukemic cells within 28 weeks in all engrafted cases (Figure 1A and Figure S1). Sequential H-E staining and immunohistochemical analysis of ALL #1 leukemic cell-engrafted humeri using anti-hCD45 antibodies demonstrated that hCD45-positive leukemic cells continued to reside within the BM (Figure 1B). Serial transplantation of leukemic cells from three representative cases into secondary and tertiary recipients demonstrated a conserved morphology (Figure 1C) and characteristic immunophenotypes (Figure 1D) in NOG mice. These results demonstrate the potential of our h-leukemic NOG model to recapitulate human ALL in NOG mice.

**Table 1:** Patient and leukemia characteristics.

Patient ID	Age/Gender	Source	WBC (/µl)	BM blast (%)	Stage	Immunophenotype	Cytogenetics
**ALL #1**	7/M	BM	183600	81.5	Diagnosis	CD45^+^CD10^+^CD19^+^CD20^-^	Normal
**ALL #2**	1/F	BM	7700	10	Relapse	CD45^+^CD10^+^CD19^+^CD20^partial^	Normal
**ALL #3**	4/F	BM	10500	91	Diagnosis	CD45^-^CD10^+^CD19^+^CD20^-^	Hyperdiploid
**ALL #4**	7/M	BM	3700	84.7	Diagnosis	CD45^+^CD10^+^CD19^+^CD20^partial^	E2A/PBX
**ALL #5**	4/F	BM	800	43.2	Diagnosis	CD45^+^CD10^+^CD19^+^CD20^+^	TEL/AML-1
**ALL #6**	4/M	BM	323700	91	Diagnosis	CD45^+^CD10^+^CD19^+^CD20^-^	Hyperdiploid
**ALL #7**	9/F	BM	3800	92.5	Diagnosis	CD45^+^CD10^+^CD19^+^CD20^partial^	TEL/AML-1
**ALL #8**	2/M	BM	4300	75.4	Diagnosis	CD45^+^CD10^+^CD19^+^CD20^partial^	MLL/ENL
**ALL #9**	8/M	BM	29400	70	Diagnosis	CD45^+^CD10^+^CD19^+^CD20^partial^	Normal

M: male. F: female. BM: bone marrow. WBC: white blood cell.

### Liver pathology in the h-leukemic NOG model resembles that in leukemic patients

**Figure 2:**
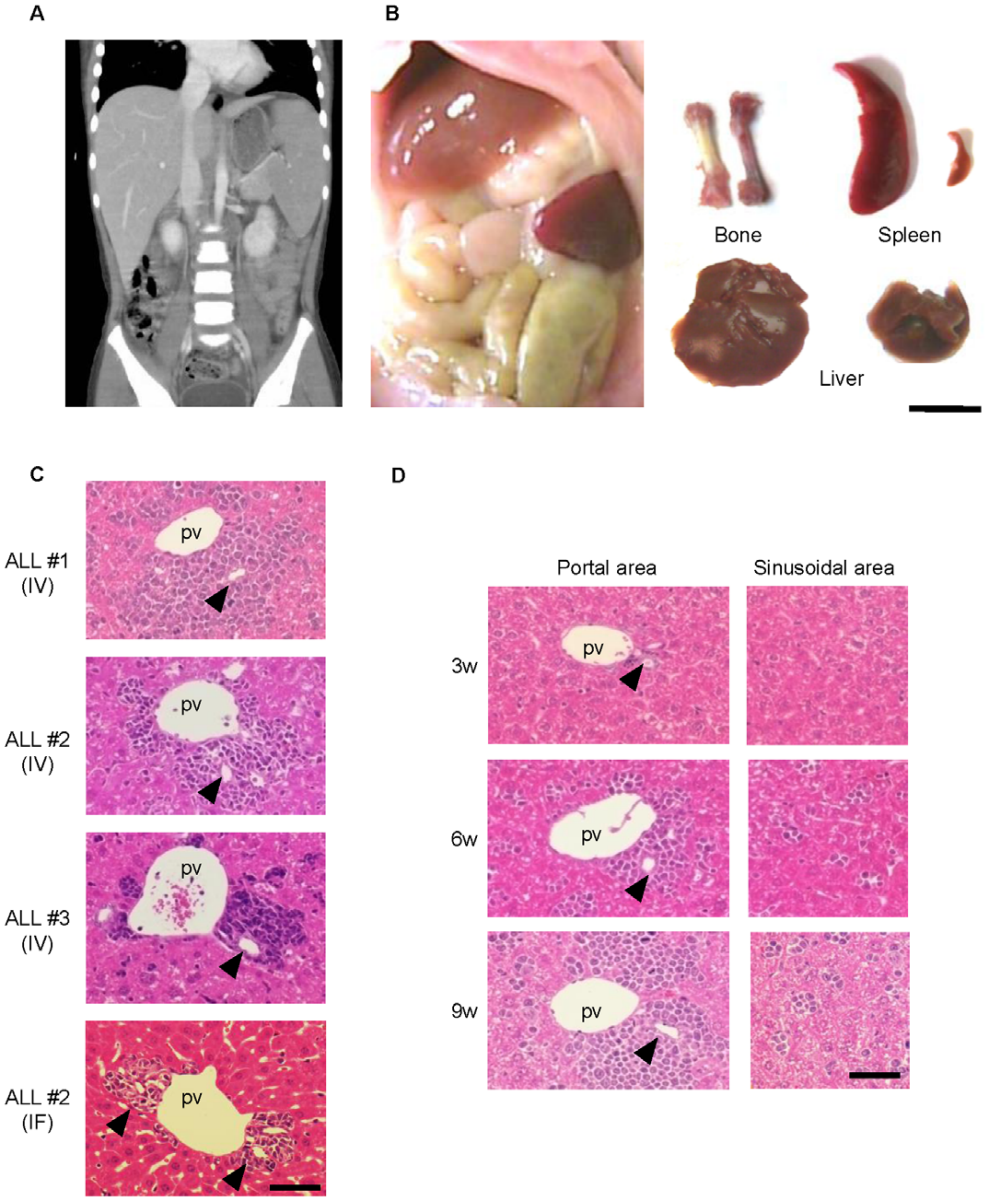
NOG recipient mice transplanted with ALL cells show a clinical leukemic pathology. (A) CT image of patient ALL#1 abdomen shows hepatosplenomegaly at diagnosis. (B) Macroscopic hepatosplenomegaly of the NOG recipient mouse transplanted with ALL#1 leukemic cells. Comparison of gross appearance of organs in normal mice (right-hand side) and leukemic NOG mice transplanted with ALL #1 leukemic cells (left-hand side). Pale femur and enlarged liver and spleen are shown. Scale bar, 1 cm. (C) In all ALL cases analyzed, the corresponding leukemic NOG recipients had large clusters of leukemic cells in portal area. Three representative cases of intra-vein injection and one case of intra-femoral injection are shown. Scale bar, 50 µm. (D) Sequential histopathological analysis of the liver of leukemic NOG mice transplanted with ALL#1 leukemic cells showed growth of leukemic cell clusters in portal areas (left column), but not sinusoidal areas (right column). Scale bar, 50 µm. pv; portal vein. IV; intra vein injection. IF; intra femoral injection. Arrowheads indicate bile ducts.

The h-leukemic NOG model showed the development of pale BM, hepatomegaly, and splenomegaly same as the original patient (Figure 2A and 2B), which was massively infiltrated with lymphoblasts (Figure S2). In the analysis of liver pathology, large clusters of leukemic cells were observed in the portal area of all cases analyzed (Figure 2C), consistent with the previously reported pathology of human ALL patients[17]. The same pathological results were obtained even after the intra-femoral injection of leukemic cells (Figure 2C).

Sequential analysis showed that small number of leukemic cells first localized around the bile ducts, and then developed into large clusters in the portal area. On the other hand, only single cells or small clusters were scattered throughout the sinusoidal areas (Figure 2D). These results suggest that our model recapitulates the histopathology of human ALL leukemic liver. Moreover, the data strongly indicate some specific mechanism by which portal areas attract and retain ALL leukemic cells.

### Leukemic cells proliferated around liver bile duct epithelial cells

**Figure 3:**
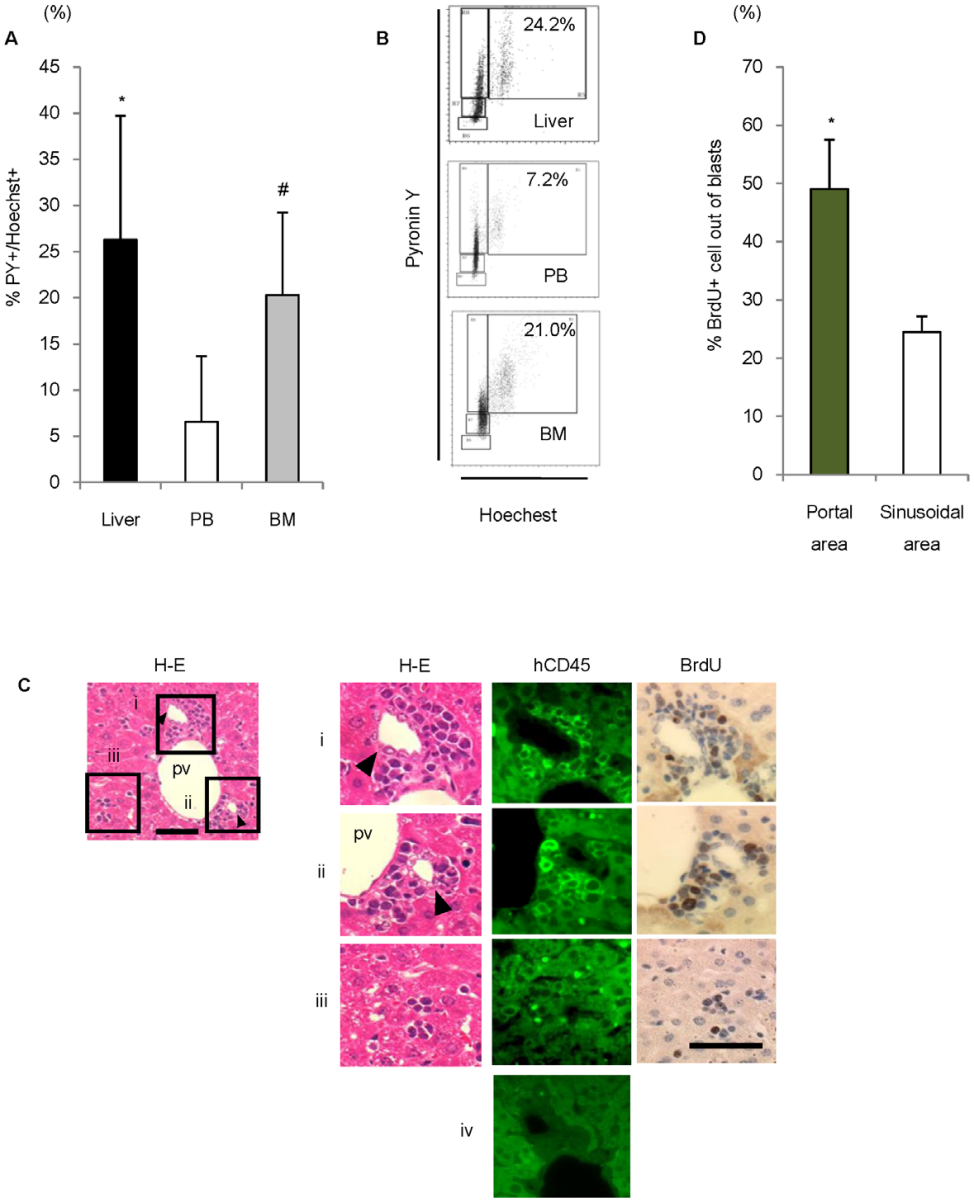
Human ALL cells preferentially proliferated around bile duct epithelial cells. (A) Frequency of S + G2/M phase leukemic cells in each organ of the mice (*P = 0.019, ^#^ *P* = 0.036, Student's *t-*test, compared with leukemic cells in the peripheral blood, *n* = 4−5). Data are shown as mean±S.D. (B) Representative FACS panels of cell cycle analysis of leukemic cells in each tissue of the recipient mice. (C) H-E, hCD45, and BrdU staining of liver sections of h-leukemic NOG model. Bile duct epithelial cells were surrounded by large numbers of hCD45^+^ leukemic cells in portal area (i and ii). A few hCD45^+^ leukemic cells were observed as single cells or small clusters randomly distributed in the sinusoidal area (iii). Negative control (iv). Scale bar, 50 µm. pv; portal vein . Arrowheads indicate bile ducts. (D) Parcentage of BrdU-positive cells among blast cells in the portal and sinusoidal areas (*P = 0.0015, Student's *t-* test, *n* = 4). Data are shown as mean±S.D.

To confirm whether leukemic cells are proliferating after settling in the liver, we performed cell cycle analysis of leukemic cells harvested from the liver, PB, and BM. The liver and BM contained a substantial fraction of proliferating leukemic cells (Figure 3A and 3B), while leukemic cells in the PB were predominantly non-proliferating. *In vivo* immunohistochemical staining with BrdU also showed that human CD45- positive leukemic cells in the liver were in the proliferation phase (Figure 3C). Interestingly, significantly increased number of BrdU positive leukemic cells were present in the portal areas compared to that in the sinusoidal areas (Figure 3D). Together, these results suggest that leukemic cells surrounding the bile ducts are not only anchored by bile duct epithelial cells, but also undergo proliferation around the bile duct epithelial cells.

### The number of CXCR4 positive leukemic cells was significantly higher in the liver. Detailed description of liver pathology revealed the existence of a functional niche mediated by SDF-1/CXCR4 axis

**Figure 4:**
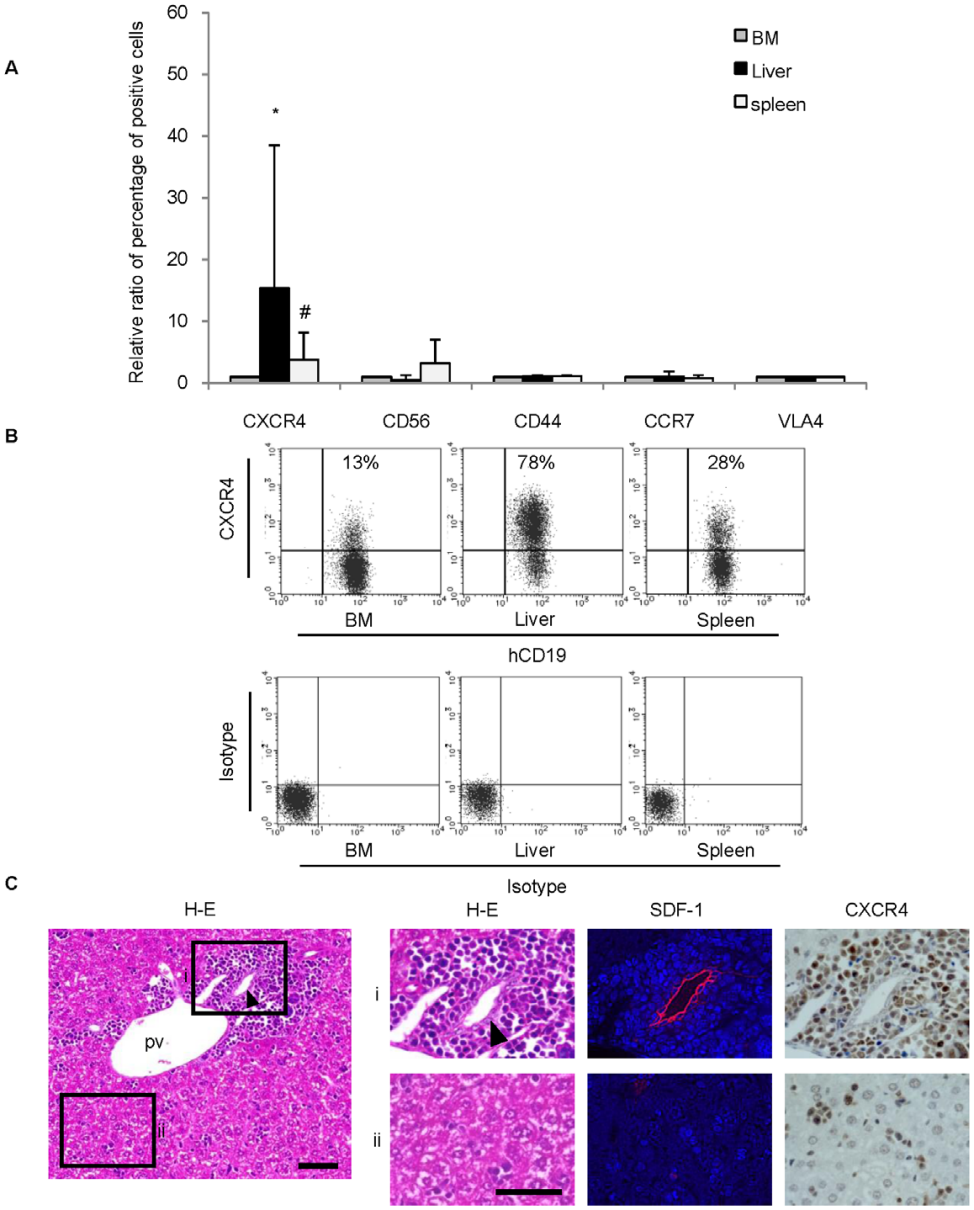
The ratio of CXCR4-positive leukemic cells was significantly higher in the liver. Bile duct epithelial cells were strongly positive for SDF-1 and surrounded by CXCR4 positive leukemic cells. (A) Relative ratio of percentage in positive cells of CXCR4, CD56, CD44, CCR7, and VLA4 out of leukemic cells harvested from liver and spleen compaired with leukemic cells from BM (*P = 0.0099, ^#^ *P* = 0.0091, paired *t*-test. *n* = 4−9 mice per case. ALL#1, #2 and #3 were analyzed). Data are shown as mean±S.D. (B) Representative FACS panels of CXCR4 expression in leukemic cells from each organ. Leukemic cells in the liver show higher levels of CXCR4 expression than those in the BM. (C) Large number of leukemic cells were observed in the portal area, and bile duct epithelial cells are strongly positive for SDF-1. CXCR4-positive leukemic cells were present in large numbers around bile ducts. Portal area (i) and sinusoidal area (ii). Scale 50 µm. pv; portal vein. Arrowheads indicate bile ducts.

Previous studies have demonstrated that several adhesion molecules and chemokines, including CXCR4, CD56, CD44, CCR7, and VLA-4, contribute to the harboring and proliferation of leukemic cells in the BM niche and may enhance niche-mediated resistance in leukemia [21], [22], [23], [24], [25]. To identify molecules involved in the pathology of the ALL leukemic liver, we analyzed their expression on leukemic cells harvested from the BM, liver, and spleen, in the three cases listed in Table 1. Flow cytometric analysis revealed that the percentage of CXCR4-positive leukemic cells was significantly higher in the liver than that in other organs (Figure 4A and 4B). Consistent with previous studies [15], [26], [27], SDF-1 was expressed on bile duct epithelial cells in the portal area of the h-leukemic NOG model (Figure 4C). Immunohistochemical analysis showed that CXCR4 positive leukemic cells were mainly distributed in the portal area, surrounding SDF-1 positive bile duct epithelial cells, but not in the sinusoidal area (Figure 4C). Moreover, proximity ligation assay confirmed the specific interaction between CXCR4 on leukemic cells and SDF1 (Figure S3). Therefore, these results strongly suggest that the SDF-1/CXCR4 axis plays a role in the hepatic niche of leukemic cells.

### The SDF-1/CXCR4 axis stimulates not only migration, but also proliferation, of ALL leukemic cells *in vitro* and *in vivo*

The SDF-1/CXCR4 axis is a key factor in the migration and proliferation of various cells, including neoplastic cells *in vivo* [18], [19], [28]. Thus, we sought to directly examine the influence of the SDF-1/CXCR4 axis on leukemic cell migration and proliferation.

**Figure 5:**
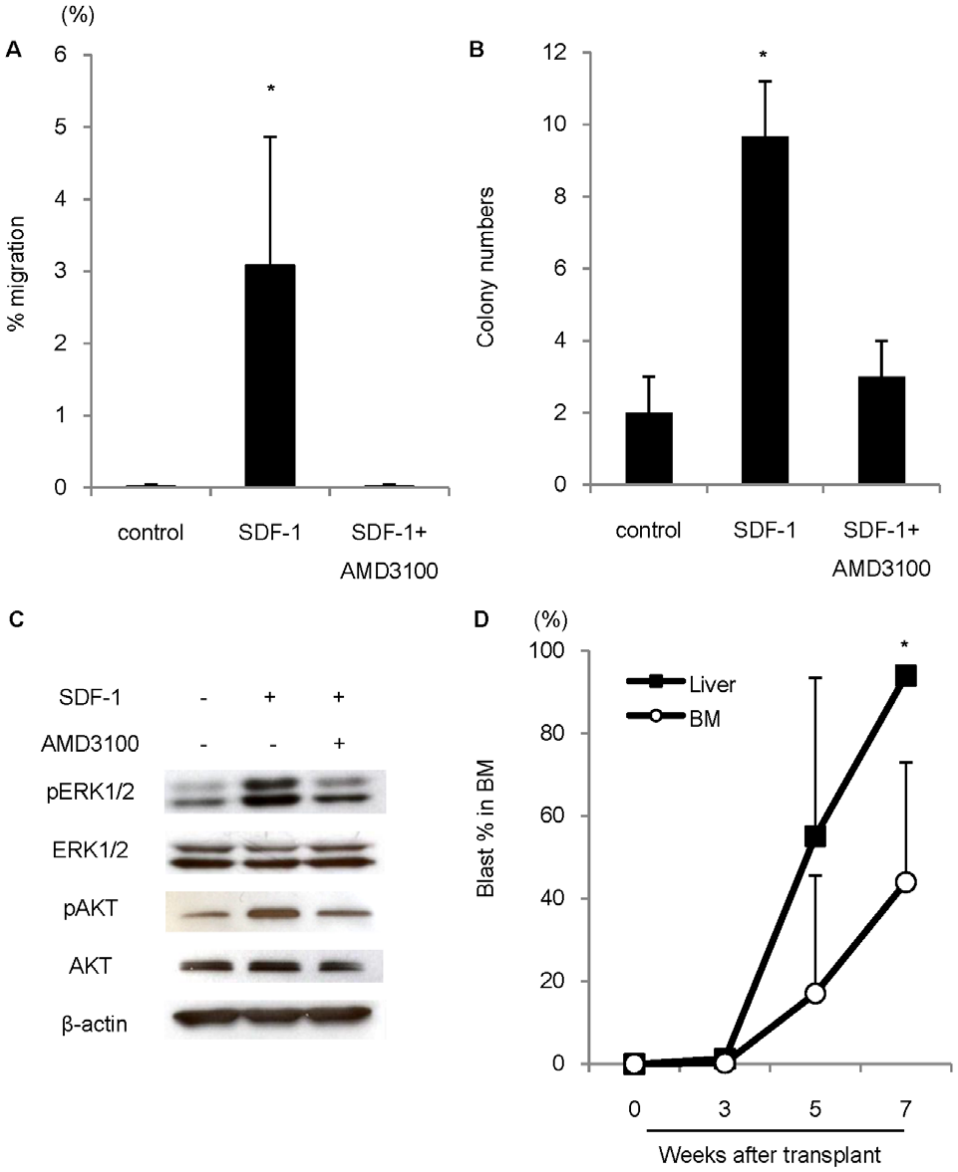
The signaling via SDF-1/CXCR4 axis stimulates migration and proliferation of leukemic cells. (A) Leukemic cells were plated onto upper chamber of transwell plates with 250 ng/ml SDF-1α in lower chamber, in presence or absence of AMD 3100 pretreatment. Results expressed as percentage of migrating cells to input cell number. (*P = 0.014, Student's *t-* test, *n* = 4). (B) Methylcellulose colony-forming assay to evaluate effect of SDF-1/CXCR4 signaling on leukemic cell proliferation. Addition of SDF-1α significantly increased leukemic colony numbers, and treatment with AMD3100 counteracted effect of SDF-1α (*P = 0.0019, Student's *t-*test, *n* = 3). (C) Phosphorylation of ERK1/2 (pERK1/2) and Akt (pAkt) was detected by Western blot analysis. β-actin was used as a loading control. (D) Sequential FACS analyses showing bone marrow engraftment after transplantation of leukemic cells harvested from BM and liver of ALL leukemic cell-transplanted mice. Graphs show percentage of blast cells in recipient BM (*P = 0.0048, Student's *t -*test, *n* = 4−8 mice per case). Data are shown as means±S.D.

First, we performed a chemotaxis assay by stimulating CXCR4 with its ligand SDF-1 (Figure 5A). Leukemic cells harvested from the liver migrated avidly in response to SDF-1, and this migration was suppressed in the presence of AMD3100, a bicyclam molecule that antagonizes the binding of SDF-1 to CXCR4. Moreover, in a checkerboard assay, cell numbers increased along the positive SDF-1 gradient in a dose-dependent manner (Figure S4). These results confirm the effects of SDF-1 on the migration of leukemic cells.

We next performed a methylcellulose colony-forming assay to examine the effect of SDF-1/CXCR4 signaling on the proliferation of leukemic cells harvested from liver(Figure 5B). The addition of SDF-1 significantly increased the number of colonies, while treatment with AMD3100 counteracted the effect of SDF-1. Western blotting revealed that SDF-1 stimulation induced phosphorylation of ERK1/2 and AKT which are known to be the important mediators of chemotaxis[26] and proliferation[20], [29], [30] of several cell types, and these phosphorylation were suppressed in the presence of AMD3100 (Figure 5C).

Next, we transplanted leukemic cells harvested from BM and liver, with different populations of CXCR4-positive cells, into NOG mice and compared the engraftment. Rapid growth of leukemic cells was observed in NOG mice transplanted with leukemic cells harvested from the liver which contain large number of CXCR4-positive leukemic cells (Figure 5D).

Taken together, these data indicate that the SDF-1/CXCR4 axis stimulates not only migration but also proliferation of ALL leukemic cells *in vivo* and *in vitro,* and implied the importance of targeting the extramedullary microenvironment to prevent recurrence from emerging from minimal residual disease in the extramedullary microenvironment in ALL patients.

### AMD3100 inhibited the *in vivo* dissemination of leukemic cells surrounding the bile ducts after chemotherapy and which thus led to the superior survival of the h-leukemic NOG mice

**Figure 6:**
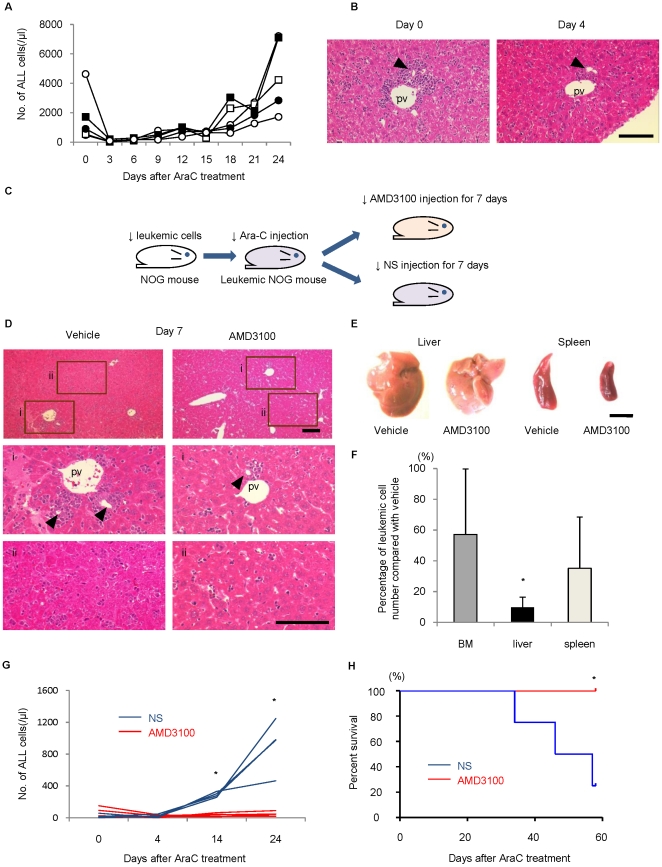
AMD3100 inhibited the *in vivo* dissemination of leukemic cells surrounding the bile ducts after chemotherapy, thus leading to the superior survival of h-leukemic NOG mice. (A) Ara-C was administered i.p. to leukemic mice, and leukemic cell counts per µl PB were monitored every three days. (B) H–E staining of leukemic liver sections before (day 0, left panel) and after (day 4, right panel) Ara-C treatment. Scale bar, 100 µm (C) Schematic of AMD3100 and normal saline (NS) injection for leukemic mice following Ara-C treatment. (D) Administration of AMD3100 resulted in reduction of leukemic cell cluster regrowth in portal areas compared with administration of NS (i).A few leukemic cells observed in the sinusoidal area in both groups (ii). pv; portal vein. Arrowheads indicate bile ducts. Scale bar, 100 µm. (E) Representative macroscopic appearance of liver and spleen in leukemic mice after AMD3100 administration. Vehicle alone group is on the left-hand side and AMD3100 injected group is on the right-hand side. Scale bar, 1 cm. (F) Percentage of leukemic cells in the BM, liver and spleen of AMD3100-treated leukemic mice compared with that of control mice. Average number of leukemic cells harvested from each organ of vehicle-injected mice was set as 100 (*P = 0.039, Student's *t -*test, compared with BM, *n* = 5 per condition).Data are shown as mean± S.D. (G) AMD3100 or NS was administered for up to 60 days to h-leukemic NOG mice after chemotherapy. The leukemic cell counts in the PB were monitored every ten days beginning 4 days after the chemotherapy. AMD3100 after chemotherapy prevented the recurrence of leukemia *in vivo* (*P*<0.01 on days 14 and 24). (H) The AMD3100-treated mice (n = 5) demonstrated a higher overall survival than the control mice receiving NS (n = 4), as estimated by the Kaplan-Meier method (*P* = 0.0221 for comparisons of the h-leukemic NOG mice treated with AMD3100 and NS).

Finally, we examined the effects of inhibiting SDF-1/CXCR4 axis on leukemic cells in the hepatic niche. A single-dose of 75 mg/kg Ara-C in h-leukemic NOG mice significantly reduced leukemic cell numbers in PB (Figure 6A). Pathological analysis of the liver showed that leukemic cells accumulating around the portal area markedly decreased after 4 days of Ara-C treatment. However only a few remaining leukemic cells were observed mainly next to bile duct epithelial cells (Figure 6B). A cell cycle analysis of leukemic cells in the liver before and after Ara-C treatment revealed that the leukemic cells in the G2/M-phase of the cell cycle were preferentially eliminated, and that this was accompanied by the enrichment of the quiescent clones after chemotherapy (Figure S5). Within 3 weeks after treatment, the number of ALL cells returned to pre-treatment levels in the PB. We used this protocol as a model for chemotherapy-induced remission and recurrence.

To elucidate the effect of SDF-1/CXCR4 axis on leukemic cell clusters regrowth in the liver portal areas, we treated h-leukemic NOG mice with Ara-C and subsequently with AMD3100 or NS for seven days (Figure 6C). In control mice receiving NS, leukemic cell regrowth in the liver was observed primarily in the portal area by day 7 (Figure 6D, left panel). In contrast, in the treatment group receiving AMD3100, leukemic cell cluster regrowth was inhibited in the portal area (Figure 6D, right panel). As a result, the macroscopic size of the liver and spleen in AMD3100-treated leukemic mice was smaller than that in control mice (Figure 6E), and leukemic cell counts and organ volumes of the liver and spleen were significantly reduced (Figure S6). Interestingly, the largest decrease in leukemic cell count was observed in the liver of AMD3100-treated mice (Figure 6F), and was seemingly correlated to the frequency of CXCR4-positive leukemic cells in each organ (see Figure 4A). During the long-term administration of AMD3100 or NS up to 60 days after AraC treatment, significantly fewer leukemic cells were present in the PB of AMD3100-treated mice compared with control mice receiving NS (Figure 6G). Consequently, the control mice lost a significant amount of body weight, while the body weight of the AMD3100-treated mice was not significantly different compared with that of normal NOG mice (Figure S7). Furthermore, the AMD3100-treated mice demonstrated a higher overall survival, as estimated by the Kaplan-Meier method (Figure 6H).

Overall, these results strongly indicate that the SDF-1/CXCR4 signaling pathway plays a crucial role in re-expansion of ALL leukemic cells in the hepatic niche after chemotherapy and provide a novel anti-leukemic therapy that targets the extramedullary microenvironment.

## Discussion

In this paper, we propose that leukemic extramedullary pathology is due to not only migrating, but also resident proliferating leukemic cells in the extramedullary niche. Using xeno-transplantation model, previous reports showed that human leukemic cells infiltrate the liver [13], [14]; however, those reports lacked pathological or molecular assessment. Here, through the analysis of h-leukemic NOG model, we have demonstrated that hepatic extramedullary microenvironments provide a niche which harbors and propagates leukemic cells.

We also demonstrated that the SDF-1/CXCR4 axis plays a crucial role in causing liver pathology. Recent studies revealed SDF-1/CXCR4 axis involvement in the development and metastasis of solid tumor [18], [19]. This axis has also been known to play an indispensable role in the homing, proliferation, and survival of both normal hematopoietic and leukemic cells in the BM niche [18], [29], [30], [31], [32], [33], [34], [35]. In pediatric ALL patients, high expression of CXCR4 in leukemic cells was strongly predictive of extramedullary organ involvement [34], which is compatible with the findings in our murine xeno-transplantation model. By analyzing the detailed structure of the hepatic niche, we found that certain extramedullary niche was also dependent on SDF-1/CXCR4 axis for recruitment and proliferation of leukemic cells.

We showed that leukemic cells in the peripheral blood were predominantly non-proliferating, although the BM and liver contain a large proportion of proliferating leukemic cells. These findings indicate that leukemic cells can proliferate efficiently in medullary/extramedullary sites if proper microenviromental conditions are provided, but cannot in the peripheral blood where microenviromental structure is absent. This finding is also compatible with the observation that *in vitro* cultures of lymphoblastic leukemic cells are more difficult to achieve in floating conditions, like in peripheral blood, than on stromal cell layers, like cells in medullary/extramedullary microenvironment [36].

In our therapeutic model, AMD3100 prevented extramedullary regrowth of leukemic cells after chemotherapy and dramatically improved the overall survival. Importantly, without AMD3100 administration, a few leukemic cells remaining in the portal region after chemotherapy appeared to contributed to the regrowth of leukemia. We speculate that two reasons may account for this extramedullary regrowth of leukemic cells. First, chemotherapy resistance may be induced by epithelial cells in the portal areas. In the BM, direct contact between ALL leukemic cells and stromal cells is one of the important mechanisms to induce drug resistance for leukemic cells [25]. Thus, it is very likely that leukemic cells next to hepatic niche cells can survive chemotherapy in an analogous manner. Second, a more fundamental reason is derived from the concept of the leukemic stem cells which have potency to regenerate leukemia [37] and contribute to relapse [5]. In acute myeloid leukemia (AML) in the BM, CD34-positive leukemic stem cells contribute to AML relapse by homing in on and expanding within the niche usually occupied by normal hematopoietic stem cells [5]. Furthermore, it has recently been shown that Ara-C treatment specifically targeted proliferating cells in an AML xenograft model, resulting in the enrichment of quiescent leukemic stem cells in the G0/G1 phase of the cell cycle, thereby contributing to disease relapse [38]. Our current data also showed that quiescent clones were not affected by chemotherapy, and thereby subsequently contribute to disease recurrence. Interestingly, during liver regeneration, the portal area serves as an important niche for both oval cells and migrating hematopoietic stem cells [10], [15], [39]. If ALL cells arise via a leukemic stem cell hierarchy similar to AML [37], this hepatic niche may serve as a haven for leukemic stem cell survival and proliferation. Considering the aforementioned reasons for the persistent presence of leukemic cells, extramedullary niche-targeting therapy could be a powerful option for preventing recurrence in extramedullary organs as well as the BM.

In conclusion, using the h-leukemic NOG model, we demonstrated that the extramedullary dissemination of leukemia is not due to nonselective infiltration, but rather systematic invasion and proliferation of leukemic cells in a particular niche at extramedullary sites. Because various signals from the leukemic niche may contribute to the progression of leukemia, future studies that elucidate the complexity of leukemic cell-host interactions will be necessary for developing novel niche-targeted therapeutics.

## Materials and Methods

### Mice

NOG mice were developed at the Central Institute of Experimental Animals (Kawasaki, Japan), as previously described[9]. All mice were kept under specific pathogen-free conditions in accordance with the guidelines of the facility.

### Human samples

BM samples were collected from patients with pediatric ALL at the time of diagnosis with written informed consent under the guidelines of the institutional ethics committee, Kyoto University Graduate School and Faculty of Medicine, Ethics Committee (approval ID is G-283). Mononuclear cells were separated by Ficoll-Hypaque density gradient centrifugation soon after aspiration.

### Primary and serial xenogeneic transplantation of ALL cells into NOG mice

Xeno-transplantation and analysis of ALL cells were performed by modifying previously reported methods[8]. In brief, leukemic cells (1×10^6^cells) were transplanted into non-pretreated 8- to 10-week old NOG mice by the tail vein or via intra-femoral injection. For serial transplantation, leukemic cells were obtained from the recipient BM and intravenously transplanted into non-pretreated NOG mice via the tail vein.

### Flow cytometric analysis of mice with transplanted leukemic cells

For analysis of leukemic cells in organs, mice were sacrificed by cervical dislocation at the indicated times after transplantation. After as much peripheral blood was collected as possible, the BM, liver and spleen were removed and mechanically dispersed. Samples from each organ were stained with antibodies after isolation of mononuclear cells. Dead cells were excluded by 4′-6-Diamidino-2-phenylindole (DAPI) staining. Samples were analyzed using a FACS LSR and Cell Quest software (Becton Dickinson) according to the manufacturer's protocol. Antibodies used for flow cytometric analysis were anti-human CD45-allophycocyanin (APC) (BD Pharmingen), anti-human N-CAM-APC (BD Pharmingen), anti-mouse CD45-APC (BD Pharmingen), anti-human CD19-phycoerythrin (PE) (eBiosciences), anti-human CXCR4-PE (BD Pharmingen), anti-human CD44-PE (BD Pharmingen), anti-human very late antigen-4 (VLA-4)-PE (BD Pharmingen), anti-human CC chemokine receptor 7 (CCR7)-PE (R&D Systems), anti-human CD45-fluorescein isothiocyanate (FITC) (BD Biosciences), and anti-human CD19-FITC (Dako).

### Histological analysis of patient and mouse samples

The histological analysis of patient samples and mouse BM was performed by May-Grunwald Giemsa staining of cytospine preparations using standard procedures. Hematoxylin-Eosin (H-E) staining was performed on tissue sections derived from the recipient mice. Detection of human leukemic cells in the murine organs by immunohistochemical analysis was performed using a previously reported method[10]. The antibodies (and dilutions) used were mouse anti-human CD45 (1∶100) (DAKO), mouse anti-human/mouse CXCL12/SDF-1α antibody (1∶100) (R&D Systems), rabbit polyclonal CXCR4 (Abcam) and Cy3-conjugated donkey anti-mouse IgG or FITC-conjugated donkey anti-rabbit IgG at a dilution of 1∶100 (both from Jackson ImmunoResearch Laboratories). Hoechst 33324 (Invitrogen) was used for nuclear staining. Prepared samples were then examined under a light or fluorescence microscope (Olympus).

### 5-bromo-2-deoxyuridine (BrdU) staining

*In vivo* BrdU labeling was performed by injecting leukemic mice with 0.2 ml BrdU (5 mg/ml, Sigma) intraperitoneally three times at 4, 24, and 48 h before analysis. Immunohistochemical staining of BrdU was performed using a BrdU staining kit (Invitrogen) according to the manufacturer's protocol.

### Cell cycle analysis

For quantification of cells in the S/G2/M phase of the cell cycle, ALL-engrafted recipient BM, peripheral blood, and liver oriented leukemia cells were labeled with Hoechst 33342 and pyronin Y, or Propidium Iodide (PI) using standard procedures. Samples were analyzed using FACS LSR and Cell Quest software (Becton Dickinson).

### Migration assay

A total of 5×10^5^ cells in a volume of 0.5 mL were added to the top chamber of 24 well MULTIWELL™ plates (Becton Dickinson). The wells separate cells using a polycarbonate membrane filter (3.0 µm pore size) from a lower compartment containing 0.5 mL RPMI with 10% FCS containing 250 ng/ml SDF-1α (R&D Systems). Chemotaxis assays were conducted at 37°C for 4 h. Some assays were performed using leukemic cells preincubated for 30 min at 37°C in the presence of AMD3100 (0.1 mg/ml) (Sigma).

### Colony assays

Colony assays were performed by modifying a previously reported method[40]. Leukemic cells were incubated in a methylcellulose culture dish at a concentration of 1×10^6^ cells/mL. Some experiments were performed on leukemic cells preincubated for 30 min at 37°C in the presence of AMD3100 (0.1 mg/ml) before the colony assays. SDF-1α (R&D Systems) was added to the control cytokines at a concentration of 250 ng/ml. Colonies were harvested, washed twice and cytospine stained with May-Grunwald Giemsa stains to identify leukemic cells.

### Western blot analysis

A total of 5×10^5^ leukemic cells were pretreated for 30 min at 37°C in the presence or absence of AMD3100 (0.1 mg/ml) and then incubated with or without 100 ng/mL SDF-1α for 5 minute. After that, cells were lysed in ice-cold lysis buffer (Thermo Scientific) supplemented with a protease inhibitor cocktail (Nacalai tesque). Lysates were then separated on a 10% polyacrylamide gel, transferred to Immobilon-P membranes (Millipore), probed with the appropriate antibodies (anti–human AKT, Ser 473–phosphorylated AKT, ERK, phosphorylated ERK , β-Actin from Cell Signaling Technologies), and visualized using an enhanced chemiluminescence (ECL) plus kit (GE Healthcare).

### Cytosine arabinoside (Ara-C) treatment and AMD3100 administration

Intraperitoneal (i.p.) injection of 75 mg/kg Ara-C (Sigma) was performed in NOG recipients transplanted with leukemic cells. AMD3100 (Sigma) was administered intraperitoneally at a dose of 200 µg for 7 days or up to 60 days in the long-term assay starting the day after Ara-C injection.

### Statistical analysis

Statistical comparisons between experimental groups were analyzed using the Student's *t*-test or paired *t*-test, and for all comparisons a *P* value less than .05 was considered significant.

## Supporting Information

Figure S1 **NOG mice support efficient human primary ALL engraftment without pre-conditioning.** Primary human ALL engraftment in NOG mice injected with 1×10^6^ BMMNCs from nine ALL patients. Percentage of human ALL cells (hCD45+ or hCD19+ cells) in recipient BM determined one to five months post-injection. Numbers in circles indicate case number and show mean percentage of ALL cells in recipient BM in each cases (*n* = 1−6 mice per case).
(TIF)

Figure S2 **BM, liver and spleen contain plenty of leukemic cells.** Pale BM, enlarged liver and spleen were massively infiltrated with leukemic cells. Histologically, no specific sites of infiltration were observed in the spleens of the transplanted NOG mice. Scale bar, 50 µm.
(TIFF)

Figure S3 **The actual interaction between CXCR4 on leukemic cells and SDF1.** Representative images of Proximity Ligation Analysis (PLA) detected by the Duolink Detection kit (Olink Bioscience, Uppsala, Sweden). The system elicits a visible signal only when the two antibodies (i.e. anti-SDF-1 and anti-CXCR4) are in close proximity. Arrows denote regions of signal amplification indicating the actual interaction between SDF-1 and CXCR4. Nuclear stain is DAPI (Blue). Scale bar indicates 50 µm.
(TIF)

Figure S4 **The checker board assay.** Increasing concentrations of SDF-1α were added to upper or lower compartments of migration chambers. Significant increases in numbers of migrating cells were observed when reagent was added to lower chambers. Baseline was defined as result of assay without SDF-1α. Increasing concentrations of SDF-1α were added to upper or lower compartments of migration chambers. Significant increases in numbers of migrating cells were observed when reagent was added to lower chambers. Baseline was defined as result of assay without SDF-1α.
(TIF)

Figure S5 **AraC preferentially eliminates cycling leukemic cells** ***in vivo.*** The cell cycle analysis of leukemic cells harvested from the liver before and after AraC treatment (80 mg/mice). The liver-oriented leukemic cells in the G2/M-phase of the cell cycle were preferentially eliminated, and the quiescent clones were not affected by the chemotherapy.
(TIF)

Figure S6 **AMD3100 inhibits hepatosplenomegaly.** Leukemic cell numbers (A) and weights of livers and spleens (B) from leukemic mice treated with vehicle alone (saline) or AMD3100 after Ara-C treatment. Each graph shows mean cell numbers and weights (* *P*<0.05. Student's *t*- test, *n* = 5 per condition). Data are shown as means±S.D.
(TIF)

Figure S7 **Administration of AMD3100 after chemotherapy prevented the recurrence of leukemia** ***in vivo*** **.** Control mice receiving NS experienced relapsed leukemia and lost significant body weight compared with AMD3100-treated mice (*P*<0.01. Student's *t*- test, *n* = 4−5 per condition) and age-matched normal NOG mice (*P*<0.01). On the other hand, the body weight of the AMD3100-treated mice was not significantly different in comparison to that of the age-matched normal NOG mice.
(TIF)
