# Supplementary figures and images for: Identification of Hepatic Niche Harboring Human Acute Lymphoblastic Leukemic Cells via the SDF-1/CXCR4 Axis
Source: PLoS One. 2011 Nov 1;6(11):e27042. doi: 10.1371/journal.pone.0027042 (PMC3206061; doi:10.1371/journal.pone.0027042)

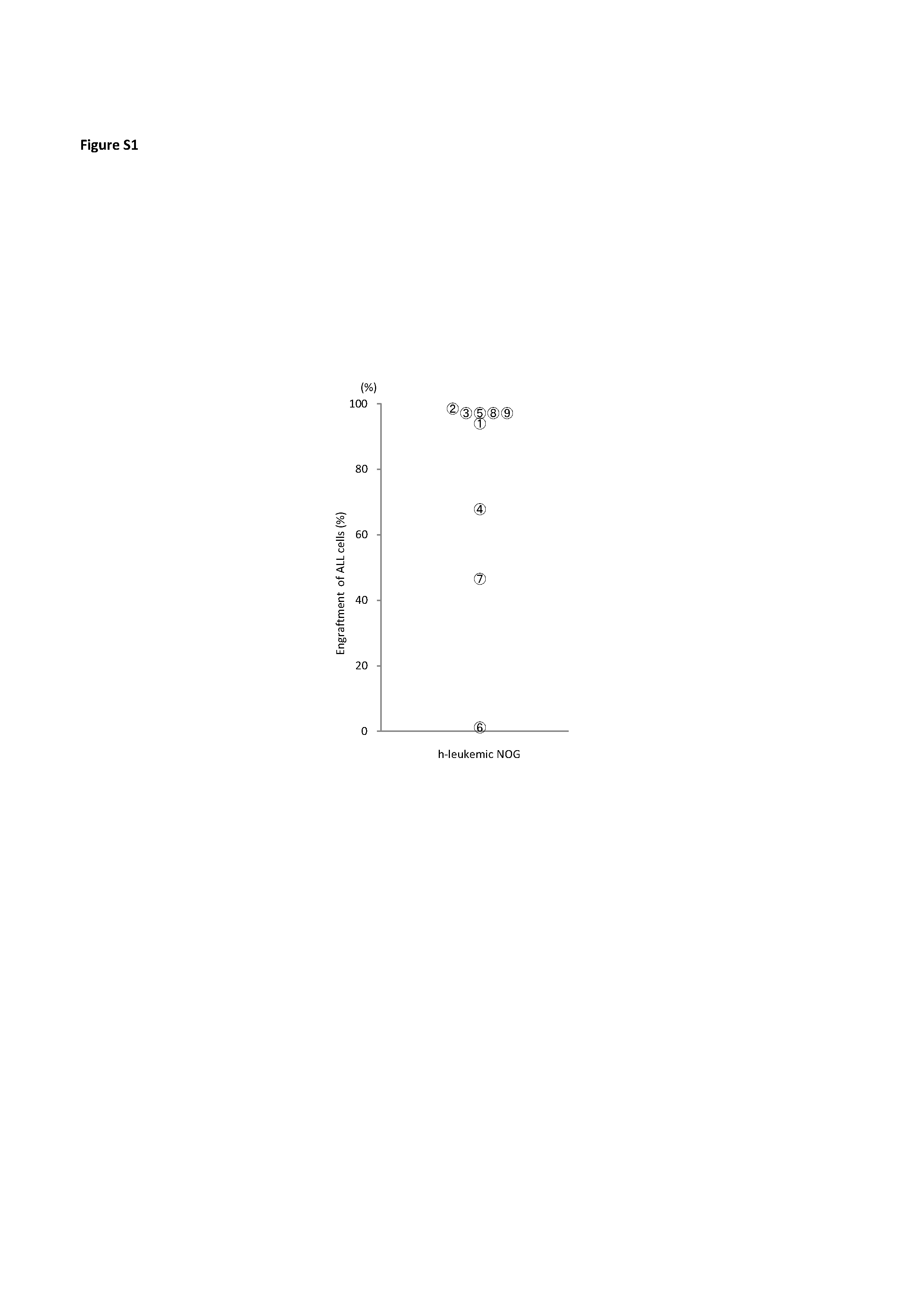

Supplement: Figure S1 — NOG mice support efficient human primary ALL engraftment without pre-conditioning. Primary human ALL engraftment in NOG mice injected with 1×10^6^ BMMNCs from nine ALL patients. Percentage of human ALL cells (hCD45+ or hCD19+ cells) in recipient BM determined one to five months post-injection. Numbers in circles indicate case number and show mean percentage of ALL cells in recipient BM in each cases (n = 1−6 mice per case). (TIF) [file pone.0027042.s001.tif]

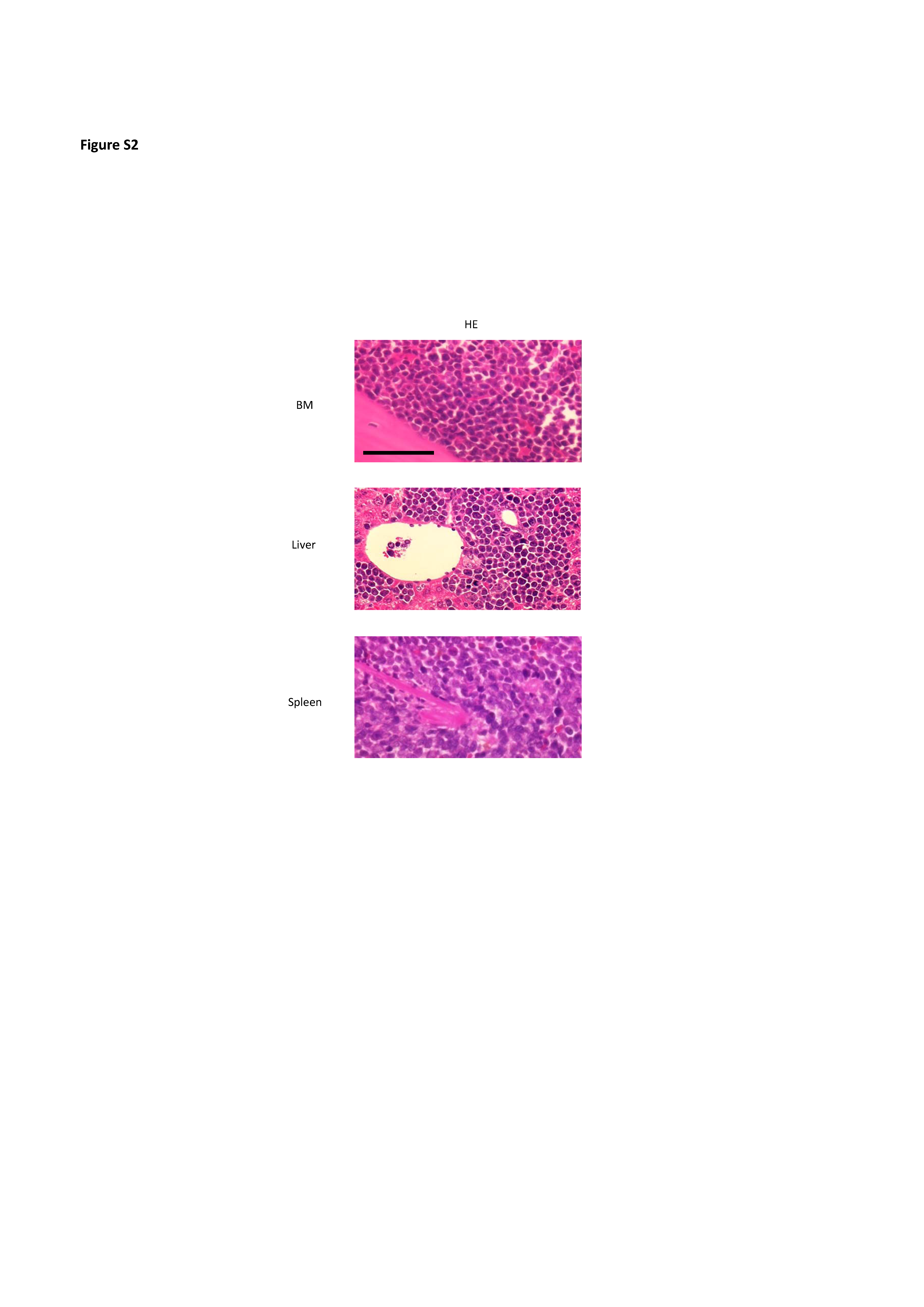

Supplement: Figure S2 — BM, liver and spleen contain plenty of leukemic cells. Pale BM, enlarged liver and spleen were massively infiltrated with leukemic cells. Histologically, no specific sites of infiltration were observed in the spleens of the transplanted NOG mice. Scale bar, 50 µm. (TIFF) [file pone.0027042.s002.tif]

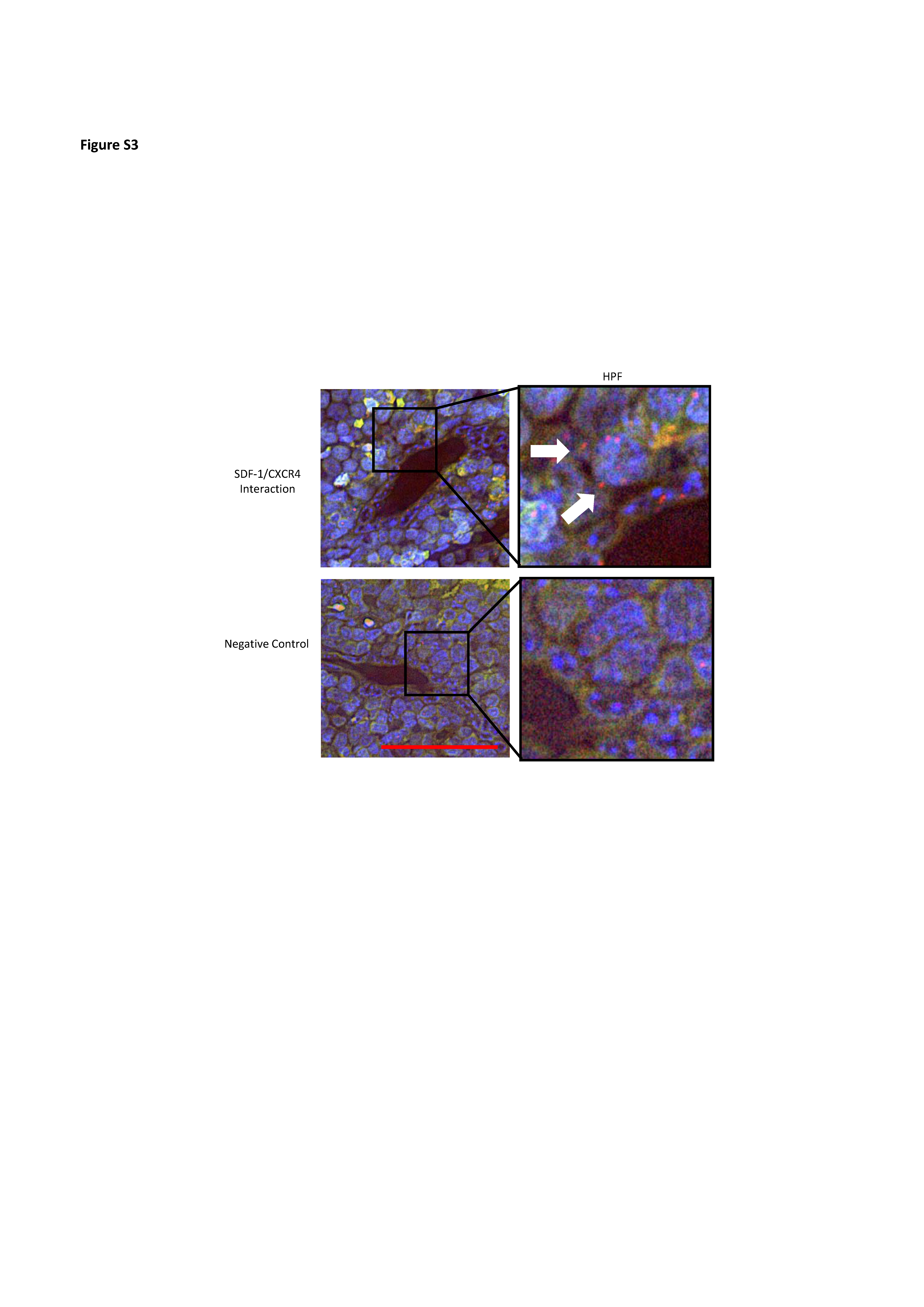

Supplement: Figure S3 — The actual interaction between CXCR4 on leukemic cells and SDF1. Representative images of Proximity Ligation Analysis (PLA) detected by the Duolink Detection kit (Olink Bioscience, Uppsala, Sweden). The system elicits a visible signal only when the two antibodies (i.e. anti-SDF-1 and anti-CXCR4) are in close proximity. Arrows denote regions of signal amplification indicating the actual interaction between SDF-1 and CXCR4. Nuclear stain is DAPI (Blue). Scale bar indicates 50 µm. (TIF) [file pone.0027042.s003.tif]

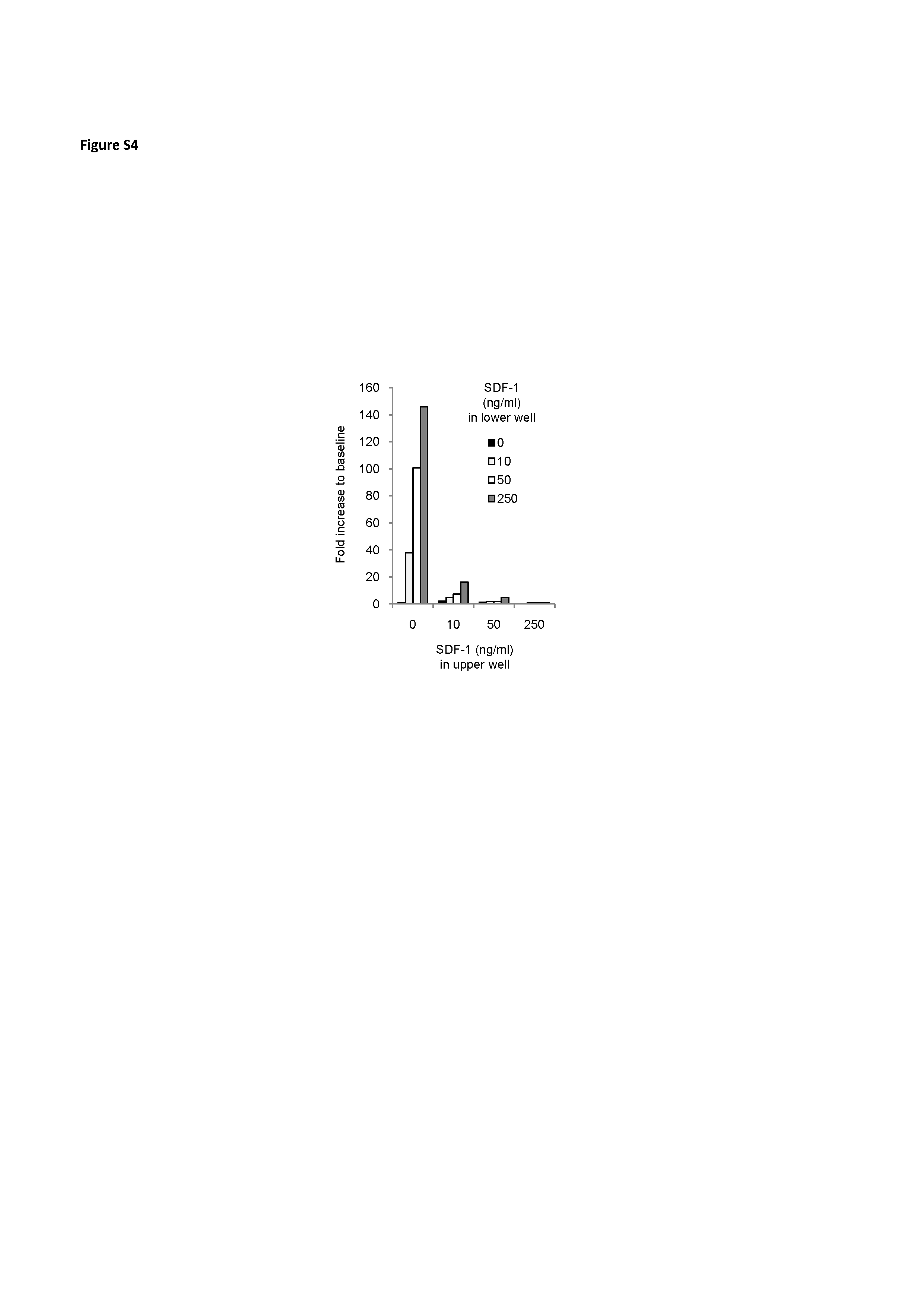

Supplement: Figure S4 — The checker board assay. Increasing concentrations of SDF-1α were added to upper or lower compartments of migration chambers. Significant increases in numbers of migrating cells were observed when reagent was added to lower chambers. Baseline was defined as result of assay without SDF-1α. Increasing concentrations of SDF-1α were added to upper or lower compartments of migration chambers. Significant increases in numbers of migrating cells were observed when reagent was added to lower chambers. Baseline was defined as result of assay without SDF-1α. (TIF) [file pone.0027042.s004.tif]

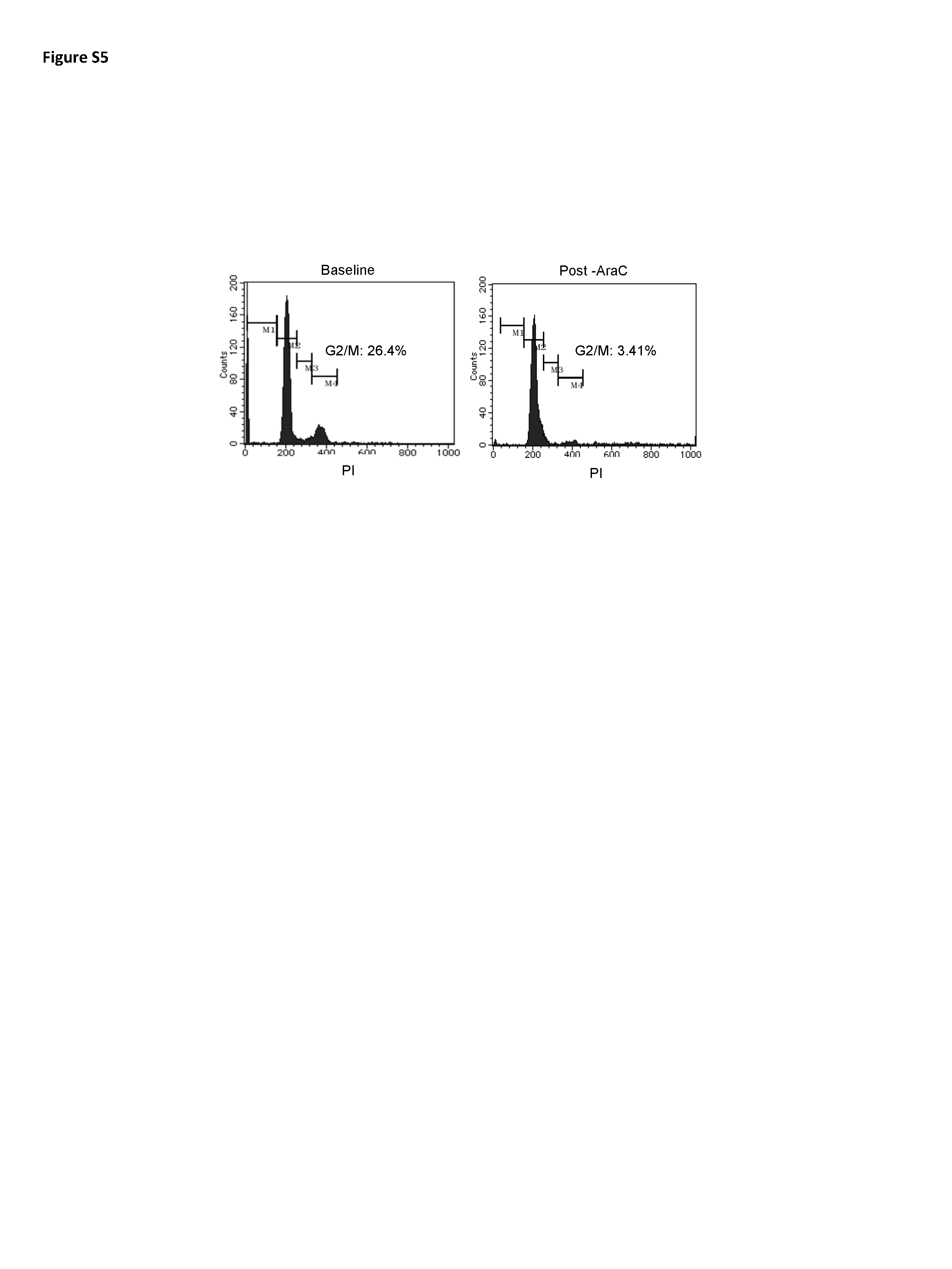

Supplement: Figure S5 — AraC preferentially eliminates cycling leukemic cells in vivo. The cell cycle analysis of leukemic cells harvested from the liver before and after AraC treatment (80 mg/mice). The liver-oriented leukemic cells in the G2/M-phase of the cell cycle were preferentially eliminated, and the quiescent clones were not affected by the chemotherapy. (TIF) [file pone.0027042.s005.tif]

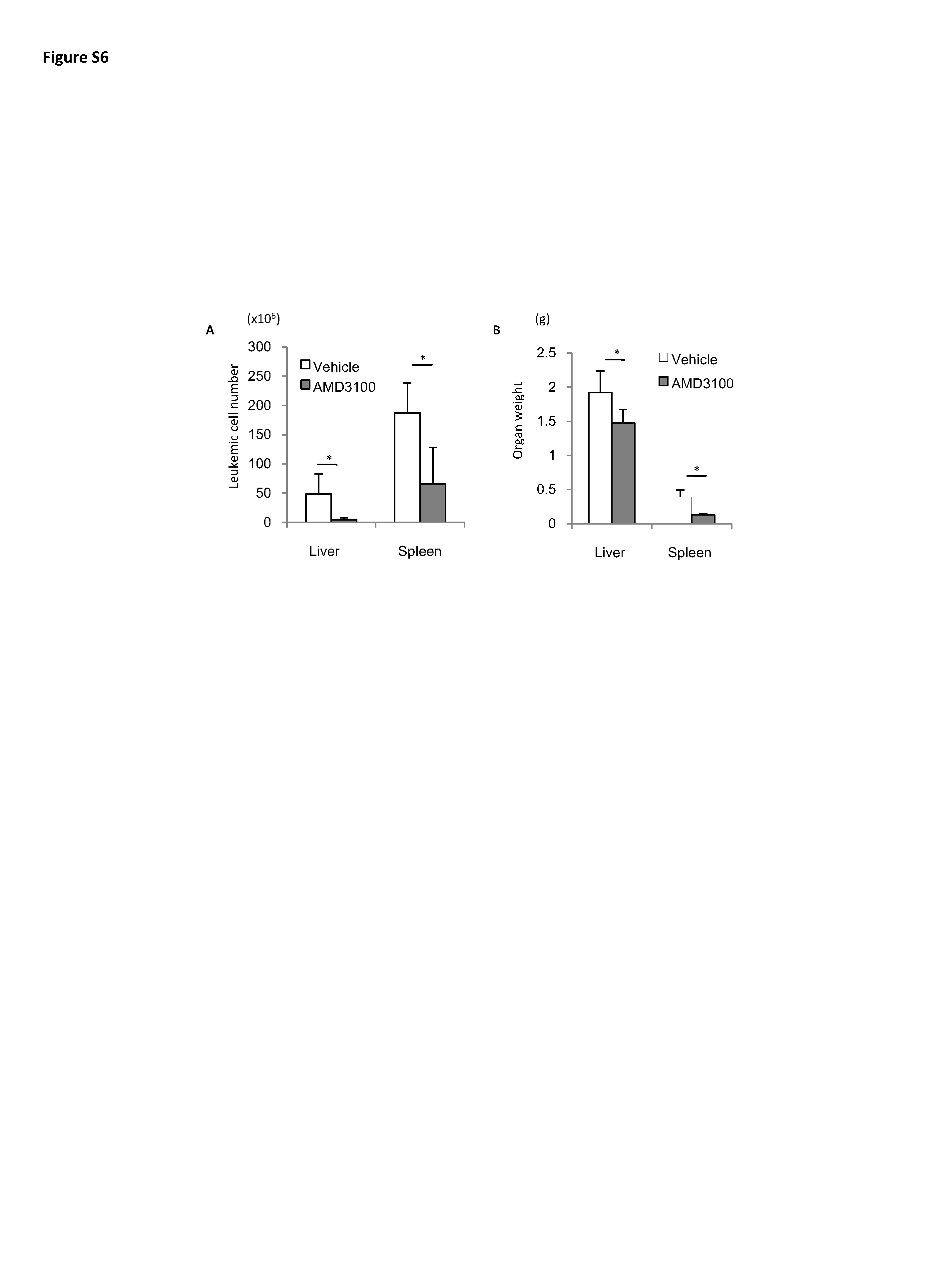

Supplement: Figure S6 — AMD3100 inhibits hepatosplenomegaly. Leukemic cell numbers (A) and weights of livers and spleens (B) from leukemic mice treated with vehicle alone (saline) or AMD3100 after Ara-C treatment. Each graph shows mean cell numbers and weights (* P<0.05. Student's t- test, n = 5 per condition). Data are shown as means±S.D. (TIF) [file pone.0027042.s006.tif]

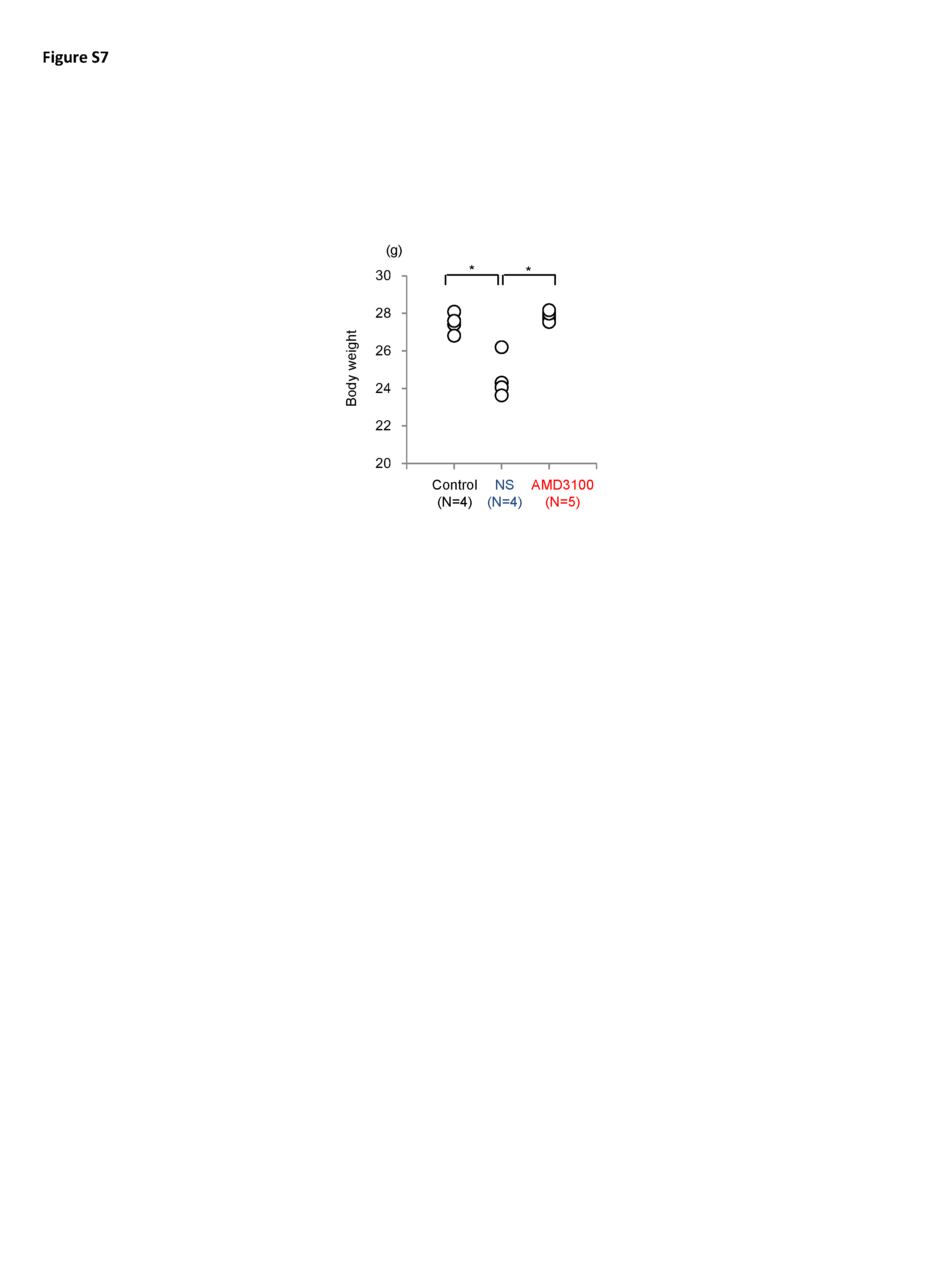

Supplement: Figure S7 — Administration of AMD3100 after chemotherapy prevented the recurrence of leukemia in vivo . Control mice receiving NS experienced relapsed leukemia and lost significant body weight compared with AMD3100-treated mice (P<0.01. Student's t- test, n = 4−5 per condition) and age-matched normal NOG mice (P<0.01). On the other hand, the body weight of the AMD3100-treated mice was not significantly different in comparison to that of the age-matched normal NOG mice. (TIF) [file pone.0027042.s007.tif]
